# Use of Terrestrial Laser Scanning Technology for Long Term High Precision Deformation Monitoring

**DOI:** 10.3390/s91209873

**Published:** 2009-12-04

**Authors:** Rok Vezočnik, Tomaž Ambrožič, Oskar Sterle, Gregor Bilban, Norbert Pfeifer, Bojan Stopar

**Affiliations:** 1 DFG Consulting, d.o.o., Pivovarniška 8, 1000 Ljubljana, Slovenia; 2 Department of Geodesy, Faculty of Civil and Geodetic Engineering, Jamova 2, 1000 Ljubljana, Slovania; E-Mails: tomaz.ambrozic@fgg.uni-lj.si (T.A.); oskar.sterle@fgg.uni-lj.si (O.S.); bojan.stopar@fgg.uni-lj.si (B.S.); 3 Geoservis, d.o.o., Litijska cesta 45, 1000 Ljubljana, Slovania; E-Mail: Gregor.Bilban@geoservis.si; 4 Institute of Photogrammetry and Remote Sensing, Vienna University of Technology, Gusshausstrasse 27-29/E122, 1040, Vienna, Austria; E-Mail: np@ipf.tuwien.ac.at

**Keywords:** terrestrial laser scanning, precise tacheometry, gnss, deformation analysis, long term monitoring

## Abstract

The paper presents a new methodology for high precision monitoring of deformations with a long term perspective using terrestrial laser scanning technology. In order to solve the problem of a stable reference system and to assure the high quality of possible position changes of point clouds, scanning is integrated with two complementary surveying techniques, *i.e.*, high quality static GNSS positioning and precise tacheometry. The case study object where the proposed methodology was tested is a high pressure underground pipeline situated in an area which is geologically unstable.

## 1. Introduction

Monitoring displacements and deformations of natural and anthropogenic spatial structures and objects represent one of the most intricate areas in geodetic surveying. The knowledge about types, characteristics and scales of structural deformations is essential when defining their nature and for the consequent verification of potential permanent damage possibilities or eventual destruction of structures. In traditional surveying, different deformation analysis approaches have evolved (e.g., Delft, Fredericton, Hannover, Karlsruhe, München, [1]). All these methods are aimed at ensuring a safe operation and usage of these structures. The second relevant aspect is closely connected with the cost-effective construction and management. The expenses of conceivable restoration may go beyond bounds; therefore, the causes for the occurrence of deformation should be discovered and prevented on time.

In recent years, terrestrial laser scanning (TLS) has become increasingly used in different engineering surveying applications, including in the field of displacement and deformation monitoring. Despite the growing number of the presented solutions, the millimetre domain in displacement detection is still a very open area of investigation. The ability to perform a rapid and dense measurement of huge amounts of object points is a tempting advantage of TLS in comparison to other sensor technologies and point-wise monitoring approaches, where deformation evaluation is limited to few discrete and well signalized points. In contrast to the lower precision of individual sampled points which may preclude their use in high precision monitoring tasks, the effective detection of deformations on the entire object covering is possible by proper modelling of the object's surfaces exploiting the high data redundancy. TLS is a remote sensing measurement technology; therefore, the direct object accessibility is not required and the influence of installation of control points or other sensor compositions onto the observed object is minimized.

In the process of long term displacement and deformation determination and analysis, the quality and stability of the chosen reference system, *i.e.*, geodetic datum, plays a vital role. The geodetic datum is realized on the basis of geodetic points which should be stabilized on geologically stable ground if deformation parameters (translations, rotations and other structural distortions, defined on the basis of the comparison of 3D surface models from TLS data) are not to be subdued by their movements. Therefore the connection of the TLS and other geodetic surveying technologies becomes inevitable. By integrating TLS with these surveying techniques into a multi-sensor composition, the weaknesses of individual measurement methods involved can be overcome, while their intrinsic advantages could be used for a complete expression of deformations on the entire surface of the structures in question.

The main contribution of this paper is the presentation of an overall and effective approach for long term high precision deformation monitoring by using geodetic measurements; namely TLS and two other point-wise surveying techniques: precise tacheometry and GNSS positioning. The primary purpose of the latter two techniques is to design and control the stability of the frame for the evaluation of point cloud displacements acquired with TLS. Besides, the paper provides conclusions regarding if and how TLS can be used in deformation detection along with other well established sensors commonly used in monitoring applications in order to assure high precision results in the long term.

The paper is structured as follows. The next subsection gives an overview of the related work and outlines its main drawbacks. In Section 2, the methodology of the proposed workflow for high precision deformation monitoring is presented. Section 3 is devoted to the description of the research which was conducted in order to evaluate the approach. In Section 4, the results of the individual techniques involved in the research are presented. Section 5 provides the analysis of the displacements computed from the acquired data. The paper finalizes with conclusions and possible future development of the proposed surveying methodology.

### Related Work

The main benefit of terrestrial laser scanning compared to other surveying techniques is the large redundancy in observations that potentially allow the detection of deformations well below the nominal individual point quality [2]. Several case studies using TLS technology for deformation monitoring have been presented in recent years. The objects of the study include dams, tunnels, bridges, towers and other buildings in general.

In [3] the authors present the results of feasibility of monitoring deformations of large concrete dams by terrestrial laser scanning. In this study it has been concluded that the stability of the reference frame is of great importance in order to separate the displacements from the noise produced by errors within the georeferencing process. Two approaches were also presented for the analysis of surface displacements, including the shortest distance between the consecutive point clouds (one being a surface model) and additionally displacements computed by comparing two regular grids of the dam face.

One interesting approach for structural monitoring of large dams by TLS is described in [4], where the Radial Basis Function was used for the parameterization of the dam surface. Moreover, the accuracy control of the georeferencing phase was performed by incorporating re-Weighted Extended Orthogonal Procrustes analysis.

In [5] it is described how artificial deformations of a cylindrical tunnel wall were detected using a statistical adjusting and testing procedure (*i.e.*, the Delft method). In this paper, the scanned surface was approximated with a cylindrical model, and the point-wise deformation analysis was performed by comparing surface patches.

Scanning of a bridge exposed to a controlled load testing is presented in [6]. The results are compared with high precision inductive transducers installed on the construction. The authors conclude that TLS is recommended as a supplementary method in load tests and displacement measurements, providing useful additional information, but cannot completely replace the traditional point-wise techniques.

Beside these case studies, many authors have applied TLS for the detection of deformations in the controlled environments or experiments with simulated values of displacements, e.g., [7]. In this way, the actual displacements and the measurement noise can be distinguished more easily, also because the effects of meteorological conditions can be neglected. Furthermore, the quality and stability of the reference frame also is not particularly addressed in these studies (it is assumed to be stable) mainly due to the fact that complementary surveying technologies must be implemented in the measurement setup in order to tackle the problem of datum correctly, which is the case in the following paper.

## 2. Methods

### 2.1. General Workflow

The primary objective of the paper is to challenge the hypothesis which states that the millimetre precision in displacements and deformation monitoring can be achieved in the long term perspective for objects and not only for few signalized (*i.e.*, marked) points, which is a typical approach when using the point-wise surveying techniques. In general, the workflow of the proposed approach can be divided into the following six steps:

1. A network of reference points should be established.
2. A geodetic network should be designed near the object of the study.
3. TLS should be performed by taking good care of the object coverage.
4. The object shape must be modelled with appropriate surfaces.
5. The object model is to be reduced to single, representative points.
6. On the basis of representative points, different deformation analysis approaches can be applied.

Apart from the presented workflow, the calibration of the instruments involved must be taken under consideration. However, as described in [8,9], the investigation of the stability of scanner systematic errors still remains somewhat open for discussion. Finally, the field work has to be performed with the utmost precision and care whereas special emphasis should be put on establishing the same surveying conditions in all measurement campaigns and following the same data processing algorithms. The surveying conditions do not include meteorological parameters since they cannot be controlled. The presented measurement approach enables a complete and effective control over the individual segments involved as well as the error propagation process. In the following sub-sections the above mentioned steps are discussed in more detail.

### 2.2. Reference Points

In order to control the quality and stability of the reference frame, the GNSS observations represent one powerful tool since currently they are the only time continuous geometric geodetic observation technology that provides absolute positions in a well defined geocentric reference system. It is limited to open terrain areas where the interruption of satellite signals can be prevented. For high precision tasks, the planning and processing strategy of GNSS observations should be based on recommendations for high precision coordinate estimation found in e.g., IGS processing strategy [10], EUREF guidelines for EPN Analysis Centres [11], or high precision geodynamic research [12,13]. The purpose of GNSS observations is therefore the realization of a stable reference frame for further terrestrial observations in all measurement campaigns.

Another possibility of controlling the reference frame is to use precise tacheometry; however, in this case the reference points must be checked for their quality and stability according to one of the methods mentioned in [1], with further consideration of field work recommendations from Section 2.3. If tacheometry is used in this step, it is important that there are enough reliable orientation points in the line of sight.

In general, this part of the workflow is one of most elusive ones to be performed in the long term perspective, depending particularly on the site characteristics.

### 2.3. Geodetic Network

The reference frame is linked with the TLS measurements (*i.e.*, point clouds, acquired in step 3) on the basis of the reference points forming the geodetic network. Therefore, this network must include the reference points, scanner target positions and control points as well. The control points can be utilized for comparison with the TLS results or may support the determination of the representative points described in Section 2.6.

It is important to design a high quality network with appropriate configuration near the object of the study to be used for an accurate relative orientation of adjacent point clouds. In high precision surveying, this task is commonly a domain of precise tacheometry. For the estimation of high precision coordinates of network points, the tacheometric measurements are usually performed in several sets of angles, measuring horizontal and vertical angles and slope distances. Many precise electronic tacheometers provide the ATR (Automatic Target Recognition) functionality which can be used to minimize the observer-related errors and to speed up the measurement process. This way a high measurement redundancy can be achieved in order to assure the quality and stability of coordinate estimation. However, if ATR is applied, the standard deviations of raw measurements have to be examined to exclude the presence of gross errors which may occur due to the automatic measurement process (e.g., in case, when two reflectors are located almost in line).

Finally, the measured slope distances have to be corrected properly for all errors which may systematically affect the measured quantities. The purpose of these corrections is to estimate the unknown coordinates of network points only on the basis of measurements affected by random errors. In order to perform these corrections, the atmospheric conditions have to be taken into account. A detailed description of slope distance corrections can be found in the literature, e.g., in [14].

### 2.4. Terrestrial Laser Scanning

As already mentioned in Section 2.1, the TLS measurements should result in sufficient object coverage, *i.e.*, point cloud density. Moreover, the point density depends not only on the predefined scan parameters (angular resolution) but also on the scanning geometry, *i.e.*, the incidence angle and distance to the object. The selection of these parameters has a direct influence on the quality of the point clouds. In [15], the authors conclude that by simply moving the scanner by two meters the point cloud quality can be improved by 25%. The effects of the object surface orientation on the quality of the data have also been studied in e.g., [16].

Eventually, the object coverage also depends on the selection of instrument (scanner) stations. The individual point clouds have to be registered in one common reference coordinate system. The quality of the relative orientation of scans is closely connected with the proper configuration of scanner targets in the geodetic network described in Section 2.3 as well as their precision parameters.

### 2.5. Modelling the Object Shape

The intrinsic character of TLS can be thoroughly exploited in the phase of object shape modelling. The object shape must be modelled with appropriate surfaces, including discontinuities, e.g., edges, break lines, *etc*. There are a number of ways in which surfaces can be represented, ranging from geometric primitives, such as planes, cylinder or spheres, to more complex ones, such as parametric patches and NURBS (Non Uniform Rational Basis Spline), which may be more convenient in case of more complex objects with more surface features. The selection of the appropriate surfaces very much depends on the object itself; however, the object model should resemble the actual shape to a required degree. In many cases the man-made objects can be modelled with geometric primitives only.

### 2.6. Determination of Representative Points

The determination of identical representative points in all measurement campaigns is of great importance in order to treat their displacements correctly. Again the definition of these points is problem dependent. If the object's shape has been deformed, the representative points must be determined on the surface itself. Contrary to that, if the object's shape has remained unchanged and it has only changed its position, the object may be presented by some specific points on its surface. For objects with well defined geometry which have not changed their form, we may choose the representative points which do not necessarily lie on the surface of the object (e.g., object axis).

### 2.7. Deformation Analysis

One common approach for the determination of displacements is to use the rule of thumb [17]. In this case, the point has moved, if the displacement vector is bigger than the positional standard deviation of end points increased by a factor of 3 or 5. Three sigmas are sometimes taken as the limit value to what can be regarded as the random error of the determined position. Therefore, any larger deviation from the estimated position is usually considered a blunder or an actual displacement.

Apart from this simple approach, there are many more sophisticated statistical algorithms for the analysis of deformations which are not discussed here.

## 3. Research

### 3.1. The Test Field and Its Characteristics

**Figure 1.**
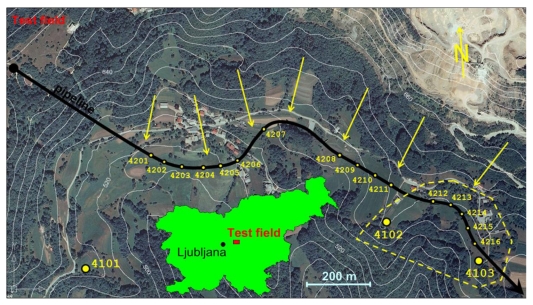
Orthophoto image of the test field (due to the size of the test field and the research nature of this research project, only the area, indicated by the dashed line, was examined).

The pipeline used in our research for the evaluation of deformations, is a part of the Slovene natural gas distribution network operated by the company Geoplin, the biggest distributor of natural gas in Slovenia. This network has been established for the transmission of gas for industrial facilities only, therefore the pressure inside the pipeline is very high (5,000 kPa). Due to the high pressure level and variations in geomorphology, some parts of the network must be monitored continuously (annually) for possible displacements and deformations. The monitoring seems even more justifiable since in many of these critical locations the pipeline runs close to permanent human settlements. For our test field, the most problematic of these locations, which is situated about 30 km east of Ljubljana, was chosen (Figure 1).

From Figure 1 it may be concluded that the pipeline orientation is approximately parallel to the contour lines, making it extremely vulnerable to perpendicular tensions of the earth masses above the level of the pipeline (indicated with arrows). The height of the terrain decreases from north to south, with an average elevation being approximately 600 m. The sliding of the ground layers becomes even more intense during periods of heavy rainfall, particularly in spring and autumn. Therefore, the pipeline manager has already built a drainage system in some parts of the area in order to draw off the excessive water volume.

**Figure 2.**
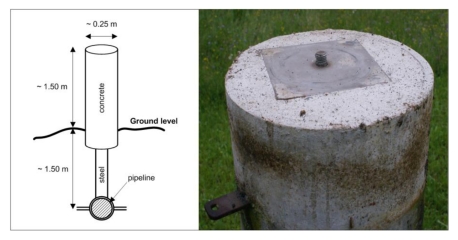
Pillars used for monitoring the movements of the underground pipeline.

At the time of the construction in the late 1970s, when the pipeline was placed underground, special concrete pillars for geodetic observations directly connected to the pipeline below were installed. In Figure 1 these observation pillars are indicated as dots numbered from 4201 to 4216. The detailed presentation of the design of the observation pillars is depicted in Figure 2. Additionally, three different locations were chosen according to the preceding geological survey of the site where reference pillars 4101, 4102 and 4103 were grounded (Figure 1). These pillars were placed on a presumably stable ground in order to function as reference points for the comparison of displacements of the observation pillars and were not connected to the pipeline. However, the stability of the reference pillars had never been tested before our research was conducted.

As shown in Figure 2, the top of the reference and observation pillars is equipped with a screw and a metal platform, with a screw not completely in line with the pillar axis, used for mounting a surveying instrument (e.g., electronic tacheometer or GNSS antenna) or a reflector. The steel bar connecting the observation pillars to the pipeline is about 8 cm in diameter and can be taken as solid, thus preventing occurrence of bending of the pillar axis when exposed to ground layer movements. The pipeline itself is also made of steel with 15 cm in diameter, which has to compensate for the tensions of the surrounding masses.

The stability of the observation pillars has so-far been investigated by means of simple tacheometric approach. The positions of the observation points were determined either from the reference pillars 4101, 4102 or 4103, assumed to be stable. The observation points are located at the intersections of the screws and the top planes of the observation pillars, about 1.5 m above the ground level (Figure 2). If these pillars are exposed to inclinations produced by the sliding of ground masses, then the displacements of observation points cannot be taken as reliable measures of the actual movements of the pipeline underground. The inability to access the underground pipeline directly has therefore contributed to the conclusion that the representative points (step 5) for the displacement analysis could only be obtained on the basis of modelling the shape of the above ground part of the observation pillars (Figure 2, left).

### 3.2. Field Work

According to the methodology presented in Section 2, two measurement campaigns were carried out, first at the beginning of June and second at the beginning of November 2008, together reflecting the period of six months of possible deformation process. Due to the openness of the area and the lack of reliable orientation points, GNSS appeared to be more convenient for the reference frame realization. The short time span available between both measurement campaigns suggested only the occurrence of very small deformations (in the millimetre domain). Therefore it was assumed that the initial hypothesis could be put to a test.

Before the second measurement campaign in September and October 2008, several periods of high rainfall further increased the possibilities of the occurrence of landslides at the site of our research. The meteorological conditions were also quite complementary, the air temperature in particular, therefore the acquisition of physical properties of the atmosphere was necessary in order to compute the atmospheric distance corrections. The detailed values of the measured meteorological parameters of both surveying campaigns are summarized in Table 1. A precise psychrometer for the acquisition of air temperature and psychrometric difference was used (with the thermometer resolution of 0.1 °C). The air pressure was obtained using barometer Paroscientific, model nr. 760-16B with the resolution of 0.01 mbar and relative precision of 0.01%.

**Figure 3.**
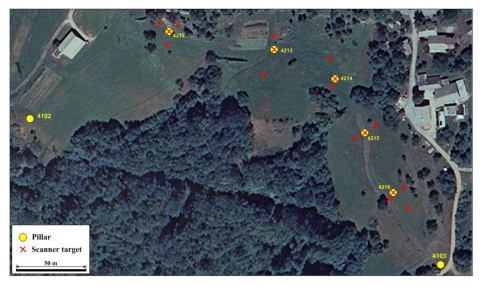
The geodetic network designed near the object of the study.

When deploying the methodology into the field, it was obvious that if the high end precision of the representative points was to be achieved, it would have taken more than one day per epoch to measure all the observation pillars from Figure 1. Because the main goal of our research was to assess the capability of the proposed methodology for the deformation monitoring purposes, the decision was made to focus only on the easternmost part of the test field (the area indicated by the dashed line in Figure 1); therefore, only five observation pillars were used in the analysis of possible displacements (pillars 4212, 4213, 4214, 4215 and 4216). However, the GNSS observations were performed on all three reference pillars 4101, 4102 and 4103 to assure better geometry and more reliable displacement estimation. The geodetic network established for the purpose of the point cloud to the reference frame connectivity and the determination of the positions of observation points located on top of each pillar is shown in Figure 3. To exclude the possible errors due to variations in the network configuration, scanner targets were placed in the same locations in both epochs.

Apart from the observation pillars (Figure 3), the scanner targets were placed onto the tripods. In the process of tacheometric measurements, also the reference pillars 4102 and 4103 were included which made it possible to compare the adjusted base distance 4102–4103 with the GNSS data.

In the geodetic network, all possible angles and distances were observed with altogether 19 instrumental stations in five sets of angles on each station, providing a high redundancy for the adjustment process, presented in Section 4.2. The Leica TCRP 1201 electronic tacheometer, which is equipped with the ATR functionality, was used. The modulation frequency, additive constant, vertical index error and collimation error were tested beforehand by the official Leica representative; therefore, the instrument was working according to the manufacturer's specifications. For the signalization of network points, the Leica reflectors GPH1P, GPR121 and GMP101 were used. The additive constants of all reflectors were also determined prior to each measurement campaign. The leveling of the instrument and reflectors was performed with precise tubular levels. After leveling all the tribrachs, they were not moved any more avoiding the occurrence of possible instrumental centering errors.

**Figure 4.**
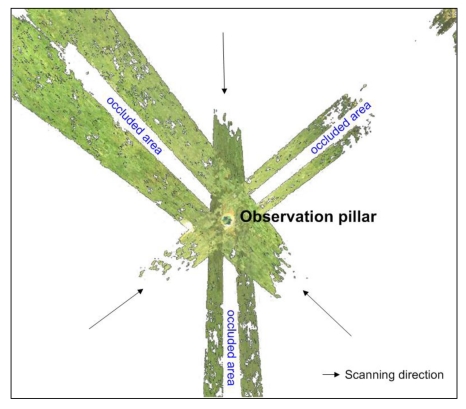
Scanner positions with respect to the observation pillars (top view). The adjacent point clouds had an overlap of approximately 30%.

The scanning was performed from three different viewing angles for each observation pillar (Figure 4), using the Leica Scanstation 2 terrestrial laser scanner, with stations regularly arranged around each pillar. The average distance from the scanner to the pillar was about 10 m with the 2 mm raster on the pillar's surface, thus resulting in a very dense point sampling of each observation pillar. Compared to the electronic tacheometer, the scanner was not calibrated before each measurement epoch. One of the main reasons is that it was provided by the official Leica representative in Slovenia and was therefore assumed to be working according to the manufacturer's specification. The processing steps applied to the TLS data are presented in Section 4.3. Altogether it took one whole day to perform the tacheometric and the TLS measurements in the field.

In each measurement campaign, the GNSS equipment was installed onto the reference pillars shortly after the tacheometric and TLS measurements were performed. The observations were carried out continuously for two/three additional days, using dual frequency Trimble 4000 SSE/SSi receivers with Trimble Compact L1/L2 with ground plane antennas or Trimble Geodetic L1/L2 with ground plane antennas. The minimum elevation angle was chosen on the basis of recommendations for high precision processing, found in [10,11] and software processing characteristics [18]. It was set to 0° to estimate troposphere parameters and height with higher reliability [19]. The sampling interval was set to 15 s. The characteristics of both campaigns are listed in Table 2.

The tribrachs on pillars 4102 and 4103 previously used for tacheometric measurements were removed only after the GNSS observations had been finished to assure no centering error was embedded when switching the instruments. In both campaigns enough GNSS observations were collected for high precision position determination.

## 4. Results

After the data acquisition phases had been completed, the next step implied the processing of the raw measurements in order to obtain the input parameters for the computation of the representative points (step 5) presented in Section 5. Each measurement technology involved was processed separately. First the coordinates of the reference pillars had to be determined on the basis of GNSS observations. These were later supplied to the tacheometric adjustment computation resulting in scanner target positions used for the registration of the point clouds as well as observation points. Finally, the pillar shape models were determined in a further adjustment procedure. The following subsections are devoted to a more detailed presentation of the data processing characteristics of the individual technologies involved.

### 4.1. GNSS

**Figure 5.**
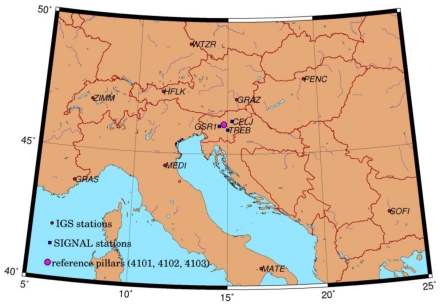
Locations of permanent GNSS stations.

Following the recommendations from Section 2.2, the realization of the reference frame in a homogenous way was ensured by tying the reference points (Step 1) to the global IGS network of permanent GNSS stations. For wider local stability of the reference frame also nearby GNSS stations from Slovene permanent network called SIGNAL [20] were included in the processing of the observations. Furthermore, it was important to decide which of these stations should be treated as reference and which as control stations. Our selection criteria were based on the station installation epoch, location and quality of stations as proposed in [12]. The permanent stations GRAS, MATE, PENC, SOFI, WTZR and ZIMM were treated as the reference stations while all others were defined as the control stations. The ITRF2005 [21] was chosen as the reference coordinate frame [22]. The locations of all permanent stations used in GNSS data processing are presented in Figure 5.

In each measurement campaign, the data was processed in one step on the basis of ionosphere free linear combination and ambiguities fixed as integer values. The software used was Bernese GPS Software, Version 5.0 [18]. The precise IGS orbits were considered [23] with corresponding Earth Orientation Parameters. Additionally, the solid Earth tides and ocean tide loading were applied as proposed by [24], with ocean tide loading coefficients obtained from Onsala Space Observatory [25] and processed according to the GOT00.2 model. Next, the GNSS antenna phase centre variations were modelled with the relative antenna calibration parameters provided by IGS. The estimation of zenith tropospheric delay was carried out according to the Saastamoinen a priori model and Niell mapping function [26] for each hour. The final solutions included modelling of tropospheric azimutal asymmetries with gradient estimation model [18] and were based on the free net estimation.

Ambiguities as the integer values were estimated on the basis of two algorithms. For short baselines (up to 150 km), the SIGMA algorithm [18] was used in two steps; initially on widelane linear combination (L5) and secondly on both carrier frequencies, *i.e.*, L1 and L2 based on widelane ambiguities. Long baselines (over 150 km) were resolved on the basis of QIF algorithm [27].

As mentioned, the final coordinates are estimated in the ITRF2005 reference frame for both campaigns. Due to tectonic motions of the Eurasian plate, the transformation of the estimated coordinates into ETRF89 was performed [28]. Finally, the 3D geocentric ETRF89 coordinates were transformed to the state planar coordinates (e.g., transverse Mercator projection) where they can be further used for the elementary surveying (Step 2). In order to assure identical coordinates in both campaigns for further processing of tacheometric and TLS measurements, a rigid translation of 4102 and 4103 station coordinates from November to June coordinate values was performed.

In Table 3, the estimated coordinates with corresponding standard deviations of the reference pillars for both campaigns on the state projection plane are given. Because heights of the network points were determined on the basis of trigonometric levelling (Section 4.2), they are not presented in Table 3. The standard deviations presented in Table 3 are the outcome of multiplying the coordinate precisions obtained by Bernese GPS Software, Version 5 processing with a factor of 10 as proposed in [29].

### 4.2. Tacheometry

By performing the tacheometric measurements in five sets of angles, the first step of the data processing phase included the computation of mean values of individual measurements. In order to properly reduce the slope distances, the meteorological distance correction factors were computed using temperature, air pressure and partial water vapour pressure provided by the psychrometric and barometric measurements. For the computation of the partial water vapour pressure, the Sprung equation was employed for the Assmann aspiration psychrometer. The saturated vapour pressure was computed according to the Magnus-Tetens equation. The standard and the actual refraction indexes of the atmosphere were determined according to [30,31]. In the process of applying the distance correction factors, only the first velocity errors were taken into account. The second velocity errors were neglected since for the distances at the range of 400 m the value of the second velocity error is approximately 10^−5^ mm.

Furthermore, the error caused by the bending of the laser ray in the atmosphere was also skipped since it accounts for about 10^−6^ mm at 400 m, which is more than the largest measured distance. The corrected distances were then reduced to the pillar level using the measured instrument/reflector height. Contrary to that, no height offset was measured when placing the instrument/reflector on the tripods. Finally, the distances were first transformed onto the reference ellipsoid surface and next onto the national cartographic projection plane. The first transformation was only possible after the determination of the height differences between all points.

For the computation of height differences, the trigonometric levelling method was utilized. The computed height differences were then adjusted according to the least squares adjustment principles, using point 4102 as a reference. In this way, the heights of the network points above sea level were obtained along with the precision parameters. According to the results, the average standard deviation of the height in the first campaign was 0.5 mm and 0.4 mm in the second. If we were to get the correct heights of the scanner targets needed for the registration of the point clouds, also the vertical offset between the targets and the reflectors would have to be taken into consideration.

The last part of the tacheometric processing involved the least squares adjustment of all of the measured and properly reduced quantities for the estimation of the planar coordinates of the network points. All angles were assigned equal weights since the conditions throughout the individual campaigns did not change substantially. Equal weights were also applied to the network distances being relatively short and measured with almost the same precision. The observations were first adjusted as a free network with the minimum trace of the cofactor matrix of coordinate unknowns. Only in this way it is possible to obtain the precision of the measured quantities independently of the network datum. Moreover, the Baarda's Data Snooping was employed for the detection of potential gross errors, providing information on the internal and external reliability of the network [32]. In the next step, the adjustment was repeated with point 4102 assumed to be stable and the direction angle from 4102 to 4103; the two reference points showed no displacements between both epochs, as it was concluded from the GNSS observations; see Section 5.1. The results of the final adjustment are shown in Table 4.

### 4.3. Terrestrial Laser Scanning

The initial phase of the TLS data evaluation included the point cloud registration process. The average global registration accuracy, including both campaigns, was *σ_R_* = 1 mm. After registration, the data not belonging to the pillar surface was manually removed from the point clouds, resulting in about 250,000 points per pillar which were then used for modelling the shape of the pillars.

**Figure 6.**
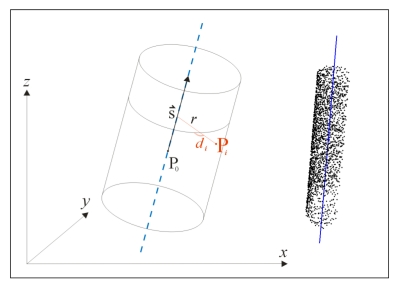
Cylinder parameters, determined in the adjustment process (left) and one example of the results in MATLAB, showing the estimated cylinder axis (right; only every 100th point is drawn).

According to Step 4, the pillars were modelled by using the cylinder model determined in the least squares adjustment process, as described in [33]. To compute the cylinder parameters, a minimum of 5 points is required, minimizing the orthogonal distances of points P*_i_*(*x_i_*, *y_i_*, *z_i_*) from the corresponding best-fit surface (Figure 6):

(1) $$d_{i}=r_{i}-r$$

where:

(2) $$r_{i}=\frac{\sqrt{u_{i}^{2}+v_{i}^{2}+w_{i}^{2}}}{\sqrt{a^{2}+b^{2}+c^{2}}}$$

and:

(3) $$\begin{array}{l} u_{i}=c\left(y_{i}-y_{0}\right)-b\left(z_{i}-z_{0}\right)\\ v_{i}=a\left(z_{i}-z_{0}\right)-c\left(x_{i}-x_{0}\right)\\ w_{i}=b\left(x_{i}-x_{0}\right)-a\left(y_{i}-y_{0}\right) \end{array}$$

Despite the fact that the cylinder is defined by:

- the point on the axis: P_0_(*x*_0_, *y*_0_, *z*_0_),
- the direction vector: $\vec{\text{s}}\left(a,b,c\right)$ and
- radius: *r*,

only five of these parameters are linearly independent (*x*_0_, *y*_0_, *a*, *b*, *r*). Since the minimization function *d* is nonlinear, the unknown parameters are computed in an iterative procedure.

**Figure 7.**
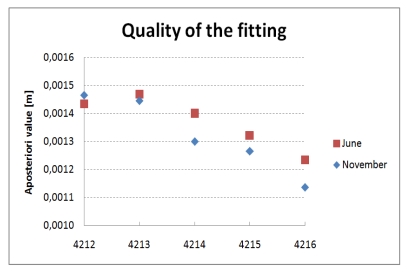
A posteriori values of cylinder fitting.

Furthermore, the validity of the models was investigated. The analysis of the quality of the fitting (*i.e.*, residual patterns) indicated that the pillar shapes are almost cylindrical with small systematic deviations below 1.5 mm. Moreover, the patterns of individual pillars were consistent in both epochs, therefore affecting the cylinder parameters in the same way. The adjustment process resulted in the average *a posteriori* value of 1.3 mm in both campaigns including all observation pillars (Figure 7). All values are well below the noise level of 2 mm stated by Leica Geosystems for the Scanstation 2 scanner. Additionally, the histograms of residuals *d_i_* indicated that no blunders were present in the data, which was also confirmed by the Gaussian probability distribution of the remaining errors.

Besides, the spatial distribution of residuals suggests that the selection of the scanner locations, as shown in Figure 4, actually compensated for the considerable errors of individual scans in the scanning direction, which is one of the downsides of the time-of-flight type scanners also employed in our research. In this way, the influences of the range inaccuracies had a more or less homogeneous effect on the computed cylinder parameters. However, there might be some temporal deviations between these distributions of the errors affecting the cylinder parameters, which was not explicitly analysed in this study.

**Figure 8.**
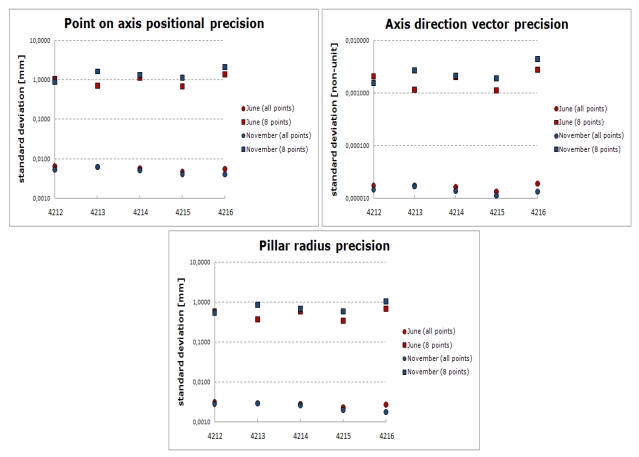
(a) The positional precision of points on the axes. (b) The axes direction vector precision. (c) Pillar radius precision. All shown in logarithmic scale.

Finally, the error propagation law was utilized for the determination of the precision measures of cylinder parameters. Almost all of the obtained standard deviations were below the order of 10 microns except for the direction cosines which were non-unit values. As mentioned in [5], the high redundancy of the data may lead to a much higher precision of the estimated parameters compared to the relatively low precision of the single point coordinates. This being a well known characteristic of TLS, it was necessary to check how realistic these precision measures actually were if we wanted to draw conclusion on deformations on their basis. To get the idea, the whole adjustment computation was repeated using only 8 points per pillar (four on the top and four on the bottom of each point cloud with equal radial arrangement). The results of both adjustment approaches concerning precision parameters are depicted in Figure 8.

The results of the two presented adjustment approaches clearly indicate the reduction of the cylinder precision parameters for the factor of 100. In this way, the upper and lower boundaries were computed, framing the quality of the adjusted quantities and offering a more firm and reliable foundation for the deformation analysis. The axes direction vectors are by far the most precisely determined parameters. Therefore, the employment of TLS has proved its value, especially when analysing only the trends in the pillar inclination in order to get a better understanding of how the terrain sliding affects the observation pillars. Last but not least, from the computed inclinations it is possible to derive the conclusions on whether the displacements of the observation points on the top of the pillars reflect the actual movements of the pipeline. In the next section, the final analysis of the computed displacements is presented, including the reference frame stability and the determination of the representative points.

## 5. Analysis and Discussion

### 5.1. The Datum Stability

**Figure 9.**
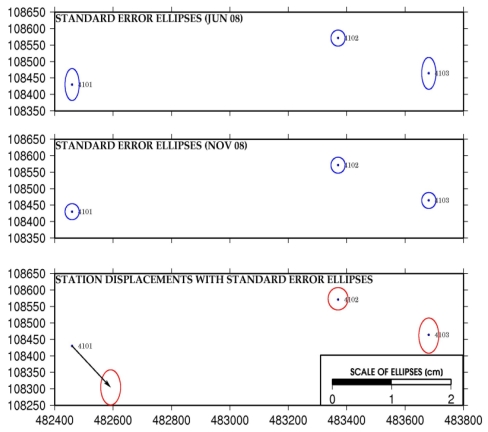
Graphical results of reference pillar displacements.

The results of the GNSS campaigns presented in Table 3 indicate that statistically identical coordinates are obtained for reference pillars 4102 and 4103. On the other hand, reference pillar 4101 shows a displacement of more than 1 cm between both GNSS campaigns. From these results it was concluded that both reference pillars 4102 and 4103 directly used in our research for further terrestrial observations and laser scanning, can be assumed as stable. In Figure 9, the analysis of the stability of the reference frame is shown graphically.

### 5.2. Determination of Representative Points

The input data for the determination of the representative points (step 5) consist of:

- the observation point locations which were treated as control points supporting the representative points computation (mentioned in Section 2.3);
- pillar axes (parameterized by points on the axes and direction vectors).

In order to assure that the points to be used for the displacement computation are actually *identical* in both campaigns, the observation pillar control points were first projected onto their axes, using the shortest distance criterion (along the perpendicular line). As mentioned before, these control points do not lie directly on the pillar axes. The orthogonal distances from the axes range from 2–16 mm, depending on the particular observation pillar.

**Figure 10.**
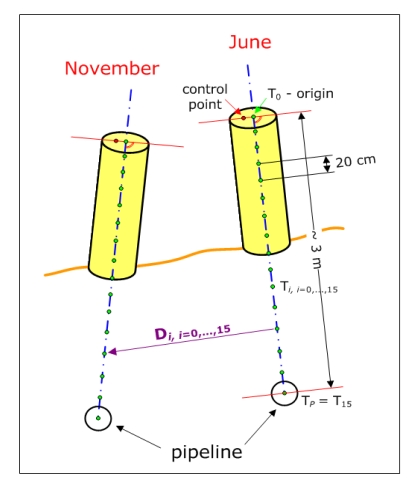
Identical points for the determination of displacements D*_i_*, including the origin T_0_ and the point in the centre of the pipeline T*_P_*.

Furthermore, all representative points were determined by extrapolating downwards to the centre of the pipeline with the help of the axes direction vectors. Additional analysis has proved that the distance of the control points from the axes had not changed. Therefore the projected points can be taken as the origins for the extrapolation. If only the pillar axes data computed from the point clouds were used, the equality of the extrapolated points could not be guaranteed because the points on the axes are not comparable. In Figure 10, the calculation of the representative points is presented together with the interpolation step of 20 cm and the maximum distance of 3 m from the origins, corresponding to the approximate distance of the pipeline centres from the pillar top ends.

### 5.3. Displacement Evaluation

**Figure 11.**
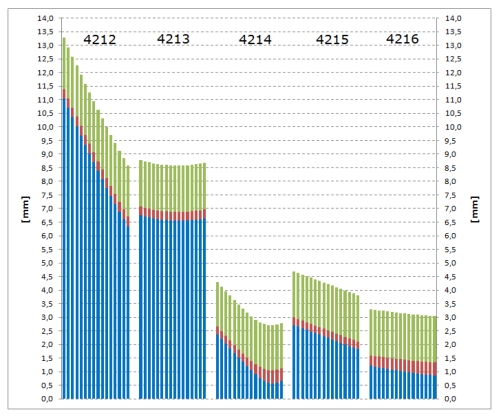
3D displacement vectors (blue bars), standard deviations of displacements (red bars) and positional standard deviations of representative points T*_i_* used for the calculation of displacement vectors (green bars, maximal values of T*_i_*_,JUN_ and T*_i_*_,NOV_ standard deviations are shown). The identical points T_0_ to T*_P_* = T_15_ go from left to right for each observation pillar.

The results of employing the approach described in the previous section have shown that pillars 4212, 4213, 4214 and 4215 have moved and pillar 4216 has not. The sizes of displacements are presented in Figure 11. The displacement of representative points on the cylinder axes is not a linear function of the distance from the corresponding origins. In case of the pillars where the shape has not changed in the period between both measurement campaigns also the analytical function of the displacements could be used to visualize the results presented in Figure 11. However, following the proposed methodology presented in Section 2.6, in case of more complex objects which deform their shape and require the representative points to be determined on the surface itself the analytical function of the displacements would be difficult if not impossible to find.

In Figure 11, for all positional standard deviations of the representative points T*_i,i_* _= 0,…,15_, the three-sigma rule was applied, expanding the confidence area up to 99.73%. This way it is clear that pillars 4212 and 4213 have been exposed to the biggest movements ranging from more than 1 cm to 6.4 mm for pillar 4212 and about 6.5 mm for 4213. By examining the trends of displacements, it is also possible to conclude that pillar 4212 has inclined, resulting in the decreasing values of displacements from top downwards. The movement of the pipeline under 4212 is consequently only 57% of the movement of the origin at the top, which means that the inclinations may have quite significant impacts on the values of displacements. Therefore, by observing only pillar peaks we cannot get accurate and reliable information on the movements of the pipeline itself. This fact is very important and may avoid or prevent false alarming from the pipeline manager side. The same inclination pattern cannot be seen for pillar 4213. The displacements indicate that all 16 points along the axis have moved almost equally and no considerable inclination effects were present.

The other two pillars, 4214 and 4215, were experiencing less impact from the ground movements, especially pillar 4214 where only the upper four points T_0_ to T_3_ have moved and the others have not. The displacements of the latter points are below the level of their corresponding end point precisions. Again, the inclination of 4214 has resulted in the reduction of displacements of about 21% when comparing T_0_ and T_3_, but no movements were detected for the pipeline centre T*_P_*.

The pillar 4215 displacements were between 2.7 and 1.8 mm, decreasing from top downwards and showing that here, too, the inclination of the pillar affected the pipeline level a little less than the top with the reduction of 33%. Yet no displacements were detected at the site of pillar 4216 since no displacement vector was larger than the corresponding end point confidence areas.

The so-far presented results were obtained by employing all TLS points, thus providing very high precision of the estimated cylinder parameters (Figure 8). By decreasing the number of points up to 50%, the same conclusions could be drawn from the displacement analysis. Hence, the presented results show a high degree of reliability with the average standard deviation of displacements of 0.4 mm. However, when applying only eight TLS points in the computation of cylinder parameters, the precision values of points T*_i_* are reduced to such an extent that the displacement pattern cannot be sustained. Additionally, the results presented in Figure 11 were also compared to the axes direction vector analysis in order to confirm the pillar inclination characteristics. Finally, the directions of the displacements were checked; *i.e.*, how they coincide with the terrain directions. Both tests have proved the quality of the results and the trends to be undisputable.

## 6. Conclusions and Future Work

The methodology and obtained results presented in the paper are clear evidence of a significant confidence for accepting the initial hypothesis. Following the methodology presented in Section 2 can lead to a high precision deformation determination in the long term for objects in question and not only for signalized (*i.e.*, marked) points. Consequently, TLS has proved to be capable of providing high precision data and therefore can be considered as a complementary surveying technique which cannot only be combined with other well established high precision surveying techniques but can also contribute to a more complete understanding of deformations. It should be emphasized that TLS is one valid solution for the displacement and deformation monitoring in 3D space. In our case, we studied the TLS applicability in determination of small changes of pillar's axes position. The inclination characteristics of the pillars could also be detected by other sensor compositions, e.g., using spirit levelling of two mutually perpendicular rigid bars mounted on top of the pillars. To ensure the stability of these platforms they would have to be permanently installed on the pillars, but the occurrence of damages cannot not be prevented since the area is not secured at all. By choosing TLS for this task, we intended to exploit the high data redundancy and the contact-less nature of this technology in order to check its sensitivity to small scale deformations without directly approaching the object itself.

In the future steps, the field work process should be optimized in order to minimize the overall acquisition time to such an extent that the millimetre level of deformations could be maintained. However, it is worth noticing that the millimetre level requires the work to be performed with a lot of care, thus preventing a radical reduction of the field work and data acquisition time. The overall time for the data acquisition and processing could be compared to any other high precision engineering task. In our research, also the high measurement redundancy in all three segments (GNSS, tacheometry and TLS) was assured, therefore finding the sufficient amount of observations seems to be reasonable as well as estimating reliable precision values for very high data redundancy. Finally, the methodological steps should be tested for other more complex objects exposed to deformations with a view to refine the individual parts if needed.

## Figures and Tables

**Table 1.** Atmospheric parameters of the two surveying campaigns (average values). All the atmospheric parameters were measured at the site of the instrument only.

**Campaign date**	**Temperature [°C]**	**Humidity [%]**	**Air pressure [mbar]**
June 2008	23.4	92.0	948.9
November 2008	−1.2	87.8	953.9

**Table 2.** GNSS campaign characteristics.

**Campaign date**	**Min. elevation angle/Sampling rate**	**Duration**
June 2008	0°/15 s	48 hours
November 2008	0°/15 s	72 hours

**Table 3.** Estimated coordinates of reference pillars.

**Pillar**	**E [m]**	***σ_E_* [mm]**	**N [m]**	***σ_N_* [mm]**	**Epoch**
4101	482,459.5975	1.0	108,430.2116	2.0	Jun 2008
	482,459.6140	1.0	108,430.1959	1.0	Nov 2008
4102	483,370.3219	1.0	108,571.3014	2.0	Jun 2008
	483,370.3219	1.0	108,571.3017	1.0	Nov 2008
4103	483,681.1483	1.0	108,464.2422	2.0	Jun 2008
	483,681.1483	1.0	108,464.2419	1.0	Nov 2008

**Table 4.** Results of the adjustment using minimum datum parameters: a posteriori variances, precisions of the adjusted observations and positional precisions of the network points.

**Epoch**	*σ̂*_0_	*σ̂_Hz_***[″]**	*σ̂_dist_***[mm]**	*σ̂_Pos,_*_max_**[mm]**	*σ̂_Pos,_*_min_**[mm]**	*σ̂_Pos,avg_***[mm]**
Jun 2008	0.99994	1.4	0.5	0.28	0.22	0.24
Nov 2008	1.00002	1.3	0.4	0.22	0.14	0.18

## References

[1] Chrzanowski A. Tasks and achievements of the FIG Working Group on deformation measurements and analysis.

[2] Gordon S.J., Lichti D.D. (2007). Modelling of terrestrial laser scanner data for precise structural deformation measurement. J. Surv. Eng..

[3] Alba M., Fregonese L., Prandi F., Scaioni M., Valgoi P. Structural monitoring of a large dam by terrestrial laser scanning.

[4] Gonzales-Aguilera D., Gomez-Lahoz J., Sanchez J. (2008). A new approach for structural monitoring of large dams with a three-dimensional laser scanner. Sensors.

[5] Van Gosliga R., Lindenbergh R., Pfeifer N. Deformation analysis of a bored tunnel by means of terrestrial laser scanning.

[6] Lovas T., Berényi A. (2009). Laser scanning in deformation measurement. GIM Int..

[7] Park H.S., Lee H.M. (2007). A new approach for health monitoring of structures: Terrestrial laser scanning. Comput.-Aided Civil Infrastr. E..

[8] Lichti D.D. (2007). Error modelling, calibration and analysis of an AM–CW terrestrial laser scanner system. ISPRS J. Photogramm..

[9] Dorninger P., Nothegger C., Pfeifer N., Molnar G. (2008). On-the-job detection and correction of systematic cyclic distance measurement errors of terrestrial laser scanners. J. Appl. Geodesy.

[10] International GNSS Service http://igscb.jpl.nasa.gov/igscb/center/analysis/.

[11] EUREF Permanent Network Guidelines for EPN Analysis Centres.

[12] Bergeot N., Bouin M.N., Diament M., Pelletier B., Régnier M., Calmant S., Ballu V. (2009). Horizontal and vertical interseismic velocity fields in the Vanuatu subduction zone from GPS measurements: Evidence for a central Vanuatu locked zone. J. Geophys. Res..

[13] Caporali A., Aichhorn C., Barlik M., Becker M., Fejes I., Gerhatova L., Ghitau D., Grenerczy G., Hefty J., Krauss S., Medak D., Milev G., Mojzes M., Mulic M., Nardo A., Pesec P., Rus T., Simek J., Sledzinski J., Solaric M., Stangl G., Stopar B., Vespe F., Virag G. (2009). Surface kinematics in the Alpine–Carpathian–Dinaric and Balkan region inferred from a new multi-network GPS combination solution. Tectonophysics.

[14] Joeckel R., Stober M. (1989). Elektronische Entfernungs- und Richtungsmessung.

[15] Soudarissanane S., Lindenbergh R., Gorte B. Reducing the error in terrestrial laser scanning by optimizing the measurement set-up.

[16] Soudarissanane S., Van Ree J., Bucksch A., Lindenbergh R. Error budget of terrestrial laser scanning: Influence of the incidence angle on the scan quality.

[17] Savšek-Safić S., Ambrožič T., Stopar B., Turk G. (2006). Determination of point displacements in the geodetic network. J. Surv. Eng..

[18] Dach R., Hugentobler U., Fridez P., Meindl M. (2007). Bernese GPS Software, Version 5.0.

[19] Even-Tzur G., Salmon E., Kozakov M., Rosenblum M. (2004). Designing a geodetic-geodynamic network: A comparative study of data processing tools. GPS Solut..

[20] Slovenian GNSS Network SIGNAL http://www.gu-signal.si/index.php.

[21] Altamimi Z., Collilieux X., Legrand J., Garayt B., Boucher C. (2007). ITRF2005: A new release of the international terrestrial reference frame based on time series of station positions and earth orientation parameters. J. Geophys. Res..

[22] International Terrestrial Reference Frame.

[23] Beutler G., Rothacher M., Schaer S., Springer T.A., Kouba J., Neilan R.E. (1999). The International GPS Service (IGS): An interdisciplinary service in support of Earth sciences. Adv. Space Res..

[24] McCarthy D.D., Petit G. IERS Conventions (2003), IERS Technical note 32.

[25] Onsala Space Observatory http://www.oso.chalmers.se/~loading/.

[26] Niell A.E. (1996). Global mapping functions for the atmosphere delay at radio wavelengths. J. Geophys. Res..

[27] Mervat L. (1995). Ambiquity Resolution Techniques in Geodetic and Geodynamic Applications of Global Positioning System. Dissertation.

[28] Boucher C., Altamimi Z. Memo: Specification for reference frame fixing in the analysis of a EUREF GPS campaign, Version 7.

[29] Mao A., Harrison C.G.A., Dixon T.H. (1999). Noise in GPS coordinate time series. J. Geophys. Res..

[30] Ciddor P.E. (1996). Refractive index of air: new equations for the visible and near infrared. Appl. Opt..

[31] Ciddor P.E., Hill R.J. (1999). Refractive index of air. 2. Group Index. Appl. Opt..

[32] Caspary W.F. (1988). Concepts of Network and Deformation Analysis.

[33] Luhmann T., Robson S., Kyle S., Harley I. (2006). Close Range Photogrammetry. Principles, Methods and Applications.

